# Muscle Ultrasound as Imaging Domain of Frailty

**DOI:** 10.3389/fmed.2022.922345

**Published:** 2022-07-11

**Authors:** Leonardo Bencivenga, Francesco Picaro, Lorenzo Ferrante, Klara Komici, Federico Ruggiero, Immacolata Sepe, Giuseppina Gambino, Grazia Daniela Femminella, Dino Franco Vitale, Nicola Ferrara, Carlo Rengo, Giuseppe Rengo

**Affiliations:** ^1^Department of Advanced Biomedical Sciences, University of Naples “Federico II”, Naples, Italy; ^2^Gérontopôle de Toulouse, Institut du Vieillissement, CHU de Toulouse, Toulouse, France; ^3^Department of Translational Medical Sciences, University of Naples “Federico II”, Naples, Italy; ^4^Department of Medicine and Health Sciences, University of Molise, Campobasso, Italy; ^5^Division of Neurology, Neurology Imaging Unit, Department of Brain Sciences, Imperial College London, London, United Kingdom; ^6^Clinica San Michele, Maddaloni, Italy; ^7^Istituti Clinici Scientifici Maugeri SpA Società Benefit, Telese, Italy

**Keywords:** frailty, ultrasound, geriatric domain, muscle thickness, Comprehensive Geriatric Assessment

## Abstract

**Introduction:**

Frailty is a geriatric syndrome, a clinical state of vulnerability for developing dependency and/or death. Due to its multidimensional nature, Comprehensive Geriatric Assessment (CGA) constitutes the best strategy to evaluate frailty in older patients. Accumulation of deficits model synthesizes the global assessment of geriatric domains in the Frailty Index (FI) score. Muscle Ultrasound (MUS) has been employed to evaluate muscle mass wasting as tool to assess sarcopenia in late life. The present study aims to evaluate the association between CGA-based FI and MUS measures in a population of hospitalized older adults.

**Methods:**

Patients aged ≥65 years underwent CGA for the evaluation of the domains of health and functional status, psycho-cognition, nutritional status, socio-environmental condition. Following standard procedure, a CGA-based FI was elaborated, taking into account 38 multidimensional items. Muscle thicknesses (MT) of rectus femoris plus vastus intermedius were measured through MUS axial cross-section. Multivariable regression analysis was employed to determine factors associated with FI.

**Results:**

The study population consisted of 136 older patients, 87 men (63.9%), with median age of 74 (70–81) years, FI of 0.3 (0.21–0.46), and MT of rectus femoris plus vastus intermedius 29.27 (23.08–35.7) mm. At multivariable regression analysis, FI resulted significantly and independently associated with age and MT.

**Conclusion:**

Muscle thicknesses of rectus femoris plus vastus intermedius, measured through MUS, resulted to be significantly related to FI in a population of hospitalized older patients. In the CGA-based assessment of frailty, MUS may constitute an additional imaging domain.

## Introduction

Frailty is a multifactorial geriatric syndrome with multiple causes and contributors, constituting the most complex expression of population aging (1). It represents a clinical state of increased vulnerability for developing dependency and/or death, due to poor homeostatic response after a stressor event, which derives from the decline in many physiological systems during the aging process. The result is a particularly susceptible substrate, on which even minor stressor events may trigger a relevant alteration in health status (2).

In the last decades, several definitions of frailty have been proposed and multiple instruments have been developed for its assessment for both clinical and scientific purposes. Although several scores have been proposed for frailty assessment, the gold standard tool has not been identified yet, thus the selection of a specific frailty instrument depends on the clinical setting, the study purpose, and the geriatric domains of interest (3). In this regard, it is important to underline that the enormous heterogeneity of the geriatric population represents a relevant difficulty in defining a single universal tool to identify frailty.

A growing piece of scientific research has focused on muscle ultrasound (MUS) to evaluate muscle mass wasting in older populations, assessing both muscle quantity and quality (4). MUS has been shown to constitute a reliable tool for the measurement of muscle size in young and old subjects, with consistent results across different body sites. Importantly, the highest intra- and inter-rater reliability has been found for large muscle groups, such as the musculus quadriceps, probably because the evaluation of smaller muscles might be complicated by limited spatial ultrasound resolution (5, 6). In this scenario, MUS has been proposed as a potential tool to assess sarcopenia in geriatric populations, including in the community setting.

Fried's frailty phenotype shows close overlap with sarcopenia, which constitutes a key contributor to frailty, facilitating the development of disability and being responsible for several adverse outcomes. Indeed, sarcopenic patients suffer increased vulnerability, negative adaptation to external stressors, and disability for basic activities of daily living (BADL) (7). Nevertheless, multidimensional frailty refers to the broader concept of complex geriatric syndrome accounting for physical, functional, mental, and social issues. Accordingly, sarcopenia should be considered as a biological substrate of physical frailty, a relevant subset of general frailty, whose assessment requires adequate diagnostic tools to reflect its multiple dimensions, such as cumulative decline in multiple body systems or functions (8).

Therefore, the central hypothesis of the present study is that MUS may constitute an additional “imaging” domain of multidimensional frailty, and to this aim, we explored the association between frailty and MUS measures in a population of hospitalized older patients.

## Methods

### Study Population

The participants have been recruited among patients aged ≥65 years referred to the Geriatric division of Department of Translational Medical Sciences of the University of Naples “Federico II”. The specific procedures of the study, described below, were performed at the resolution of the acute clinical condition that led to hospital admission. Exclusion criteria were: cachexia, extreme obesity, dialysis-dependent kidney failure and/or end-stage organ failure, central and peripheral nervous system diseases, myositis and diseases inducing muscular atrophy, major surgery on the lower limbs, and the presence of scars at the measurement sites.

All patients underwent medical history collection, clinical examination, and evaluation of the main demographic/clinical factors. The results of the main biochemical blood tests were also registered. All participants were carefully informed and signed a written consent to participate in this study. The research protocol was reviewed and approved by the Local Ethics Committee (124/17) and was conducted in compliance with the ethical principles stated in the Declaration of Helsinki.

### Frailty Assessment

All patients underwent Comprehensive Geriatric Assessment (CGA), with the evaluation of the domains of health and functional status, nutrition, psycho-cognition, and socio-environmental condition. A CGA-based Frailty Index (FI) was created following a standard procedure as proposed by the Rockwood's research group (9), taking into account a total of 38 multidimensional health deficits, such as comorbidities, laboratory and diagnostic data, and symptoms and signs of diseases (Tables 1, 2). The presence of deficits in these items was ascertained by trained physicians, each deficit was awarded 1 point if present or 0 in its absence. FI for a single participant resulted by the ratio between his/her cumulative points and the total number of evaluated items, thus this ranged between 0 and 1. A cut-off of 0.25 was used to define an individual as frail.

**Table 1:** Health variables and cut-points for the frailty index – adapted from (9).

**List of variables included in the frailty index**	**Cut point**
Help bathing	Yes = 1, No = 0
Help dressing	Yes = 1, No = 0
Help getting in/out of chair	Yes = 1, No = 0
Help walking around house	Yes = 1, No = 0
Help eating	Yes = 1, No = 0
Help grooming	Yes = 1, No = 0
Help using toilet	Yes = 1, No = 0
Help up/down stairs	Yes = 1, No = 0
Help lifting 10 lbs	Yes = 1, No = 0
Help shopping	Yes = 1, No = 0
Help with housework	Yes = 1, No = 0
Help with meal preparations	Yes = 1, No = 0
Help taking medication	Yes = 1, No = 0
Help with finances	Yes = 1, No = 0
Lost more than 10 lbs in last year	Yes = 1, No = 0
Self rating of health	Poor = 1, Fair = 0.75, Good = 0.5, V. Good = 0.25, Excellent = 0
How health has changed in last year	Worse = 1, Better/Same = 0
Stayed in bed at least half the day due to health (in last month)	Yes = 1, No = 0
Cut down on usual activity (in last month)	Yes = 1, No = 0
Walk outside	<3 days = 1, ≤ 3 days = 0
Feel everything is an effort	Most of time = 1, Some time = 0.5, Rarely = 0
Feel depressed	Most of time = 1, Some time = 0.5, Rarely = 0
Feel happy	Most of time = 0, Some time = 0.5, Rarely = 1
Feel lonely	Most of time = 1, Some time = 0.5, Rarely = 0
Have trouble getting going	Most of time = 1, Some time = 0.5, Rarely = 0
High blood pressure	Yes = 1, Suspect = 0.5, No = 0
Heart attack	Yes = 1, Suspect = 0.5, No = 0
Chronic heart failure	Yes = 1, Suspect = 0.5, No = 0
Stroke	Yes = 1, Suspect = 0.5, No = 0
Cancer	Yes = 1, Suspect = 0.5, No = 0
Diabetes	Yes = 1, Suspect = 0.5, No = 0
Arthritis	Yes = 1, Suspect = 0.5, No = 0
Chronic lung disease	Yes = 1, Suspect = 0.5, No = 0
Mini mental state examination	<10 = 1, 11–17 = 0.75, 18–20 = 0.5, 20–24 = 0.25, >24 = 0
Body mass index	See Table 2
Grip strength	See **Table 2**
Usual pace	See **Table 2**
Rapid pace	See **Table 2**

**Table 2:** Deficit cut-off values for continuous variables by sex – adapted from (9).

**Variable**	**Deficit for men**	**Deficit for women**
Body mass index (BMI)	<18.5, ≥30 as a deficit	<18.5, ≥30 as a deficit
	25– <30 as a “half deficit”	25– <30 as a “half deficit”
Grip strength (kg)	For BMI ≤ 24, GS ≤ 29	For BMI ≤ 23, GS ≤ 17
	For BMI 24.1–28, GS ≤ 30	For BMI 23.1–26, GS ≤ 17.3
	For BMI > 28, GS ≤ 32	For BMI 26.1–29, GS ≤ 18
		For BMI > 29, GS ≤ 21
Rapid pace walk (s)	>10	>10
Usual pace walk (s)	>16	>16

### Muscle Ultrasound

**Figure 1:**
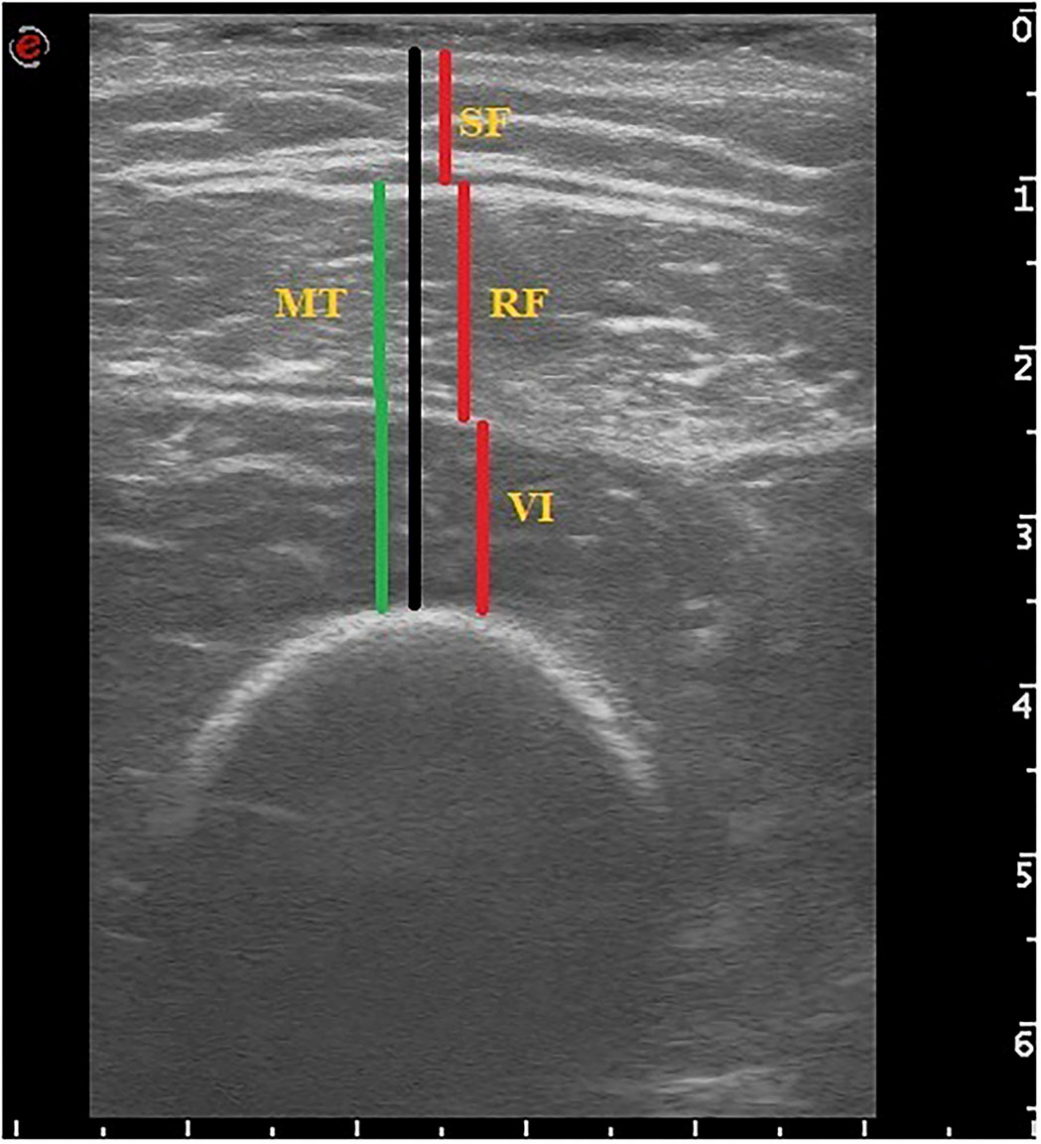
Representative image of muscle ultrasound. Muscle thickness (MT) was defined as the mean value of three measurements of the sum of the distance between the anterior fascia and the posterior fascia of the rectus femoris (RF) and the vastus intermedius (VI) muscles. SF, subcutaneous fat.

The participants were assessed in a supine position, with the knees resting in extension for 30 min. The rectus femoris and vastus intermedius of dominant thigh of each patient were assessed at mid-point between greater trochanter and proximal border of patella, following proposed standards (10). A linear array probe of an ultrasound diagnostic apparatus (MyLab™ Twice – Ultrasound Systems Esaote) was positioned perpendicular to the midpoint of the dorsal thigh to record the axial image. Ultrasound gel was applied both on the probe and the thigh to not make the two surfaces in direct contact, thus minimizing pressure on the soft tissue. Once the image was captured, thicknesses of subcutaneous fat, rectus femoris muscle, and vastus intermedius muscle were measured through axial cross-section (11). Muscle Thickness (MT) was defined as the mean value of three measurements of the sum of the distance between the anterior fascia and the posterior fascia of the rectus femoris and the vastus intermedius muscles (Figure 1).

### Statistical Analysis

Continuous variables were expressed as mean ± standard deviation (*SD*) or median and interquartile range (IQR) and compared using Student's *t*-test or the Mann–Whitney *U*-test. The Normal distribution was assessed using the Shapiro–Wilk test. The categorical variables were expressed as a percentage and compared using Pearson's χ^2^ test. Descriptive comparisons between groups were conducted according to gender and frailty status. The multivariable regression analysis was used to identify factors associated with continuous dependent variable FI. Parsimonious selection criteria were used to avoid overfitting bias. The analysis considered: age, gender, and BMI as independent variables. An alternative model was developed with subcutaneous fat thickness as an independent factor, instead of BMI. The regression model was employed to determine the impact of the MUS parameters on FI. All analyses were performed using the STATA statistical software (STATA version 17; StataCorp LLC, College Station, TX, USA), and a *p*-value < 0.05 was considered as the statistical significance threshold.

## Results

The study population consisted of 136 older patients, 87 men (63.9%), with median age of 74 (70–81) years and mean BMI of 26.01 ± 4.51 kg/m^2^. The overall sample presented MT of rectus femoris plus vastus intermedius of 29.27 (23.08–35.7) mm, and FI of 0.3 (0.21–0.46). Dividing the population according to the predetermined FI cut-off, 91 (66.9%) subjects were “frail” (FI ≥ 0.25) and 45 “non-frail.” Characteristics of the overall study population and of subgroups divided according to the frailty status are reported in Table 3.

**Table 3:** Characteristics of the overall population and of subgroup according to frailty status.

**Characteristics**	**Overall population (*n* = 136)**	**Frail FI ≥0.25 (*n* = 91)**	**Non frail FI <0.25 (*n* = 45)**	**Sig**.
Age (years)	74 (70–81)	76 (71–82)	73 (69–76)	0.007
Gender (male) *n* (%)	87 (63.9)	53 (58.2)	34 (75.6)	0.041
BMI (kg/m^2^)	26.01 ± 4.51	25.82 ± 5.08	26.38 ± 3.06	0.433
Hemoglobin (g/dl)	11.99 ± 2.43	11.81 ± 2.25	12.35 ± 2.73	0.263
eGFR (ml/min/1.73 m^2^)	65 (47–82)	64 (45–81)	66 (47–86)	0.476
Serum protein (g/dl)	6.47 ± 0.75	6.43 ± 0.76	6.59 ± 0.73	0.344
MMSE (/30)	25 (21.7–27)	24.3 (20.8–26.2)	26.7 (24.7–29)	<0.001
BADL (/6)	6 (5-6)	5 (4-6)	6 (5-6)	<0.001
IADL (/8)	7 (4-8)	6 (3-8)	8 (7-8)	<0.001
POMA (/28)	24.5 (16-27)	21 (12-26)	27 (25-28)	<0.001
SPPB (/12)	5.85 ± 3.5	4.68 ± 3.21	8.22 ± 2.83	<0.001
MNA (/30)	22 (19–24.5)	20.5 (17.5–23)	24 (23-26)	<0.001
CIRS (*n*)	3.76 ± 1.96	3.90 ± 1.84	3.47 ± 2.18	0.254
Chronic drugs (*n*)	6.71 ± 2.85	6.64 ± 2.85	6.87 ± 2.86	0.661
PASE (*n*)	80 (37.85–125)	55 (20-110)	116 (81–151)	<0.001
Social support score (/17)	6.47 ± 2.72	7.25 ± 2.52	4.89 ± 2.42	<0.001
Grip strength (kg)	24.15 ± 9.93	23.4 ± 10.46	26.67 ± 8.19	0.441
FI (/1)	0.3 (0.21–0.46)	0.4 (0.33–0.56)	0.18 (0.11–0.21)	<0.001
Rectus femoris (mm)	17.01 ± 4.65	16.1 ± 4.37	18.85 ± 4.37	0.002
Vastus intermedius (mm)	12.3 (9.1–16.05)	10.9 (8.36–15.4)	13.96 (11.6–17.5)	0.002
MT (mm)	29.27 (23.08–35.7)	26.4 (21.9–33)	33.4 (26.8–38.5)	<0.001
Subcutaneous fat (mm)	11.4 (8.16–18.05)	11.7 (8.13–18.8)	10.8 (8.4–15.1)	0.533

*BADL, basic activity of daily living; BMI, body mass index; CIRS, cumulative illness rating scale; eGFR, estimated glomerular filtration rate (according to CKD-EPI formula); FI, frailty index; IADL, instrumental activity of daily living; MMSE, mini mental state examination; MNA, mini nutritional assessment; MT, muscle thickness (vastus intermedius plus rectus femoris); PASE, physical activity scale for the elderly; POMA, Tinetti's Performance Oriented Mobility Assessment; SD, standard deviation; SPPB, short performance physical battery*.

**Figure 2:**
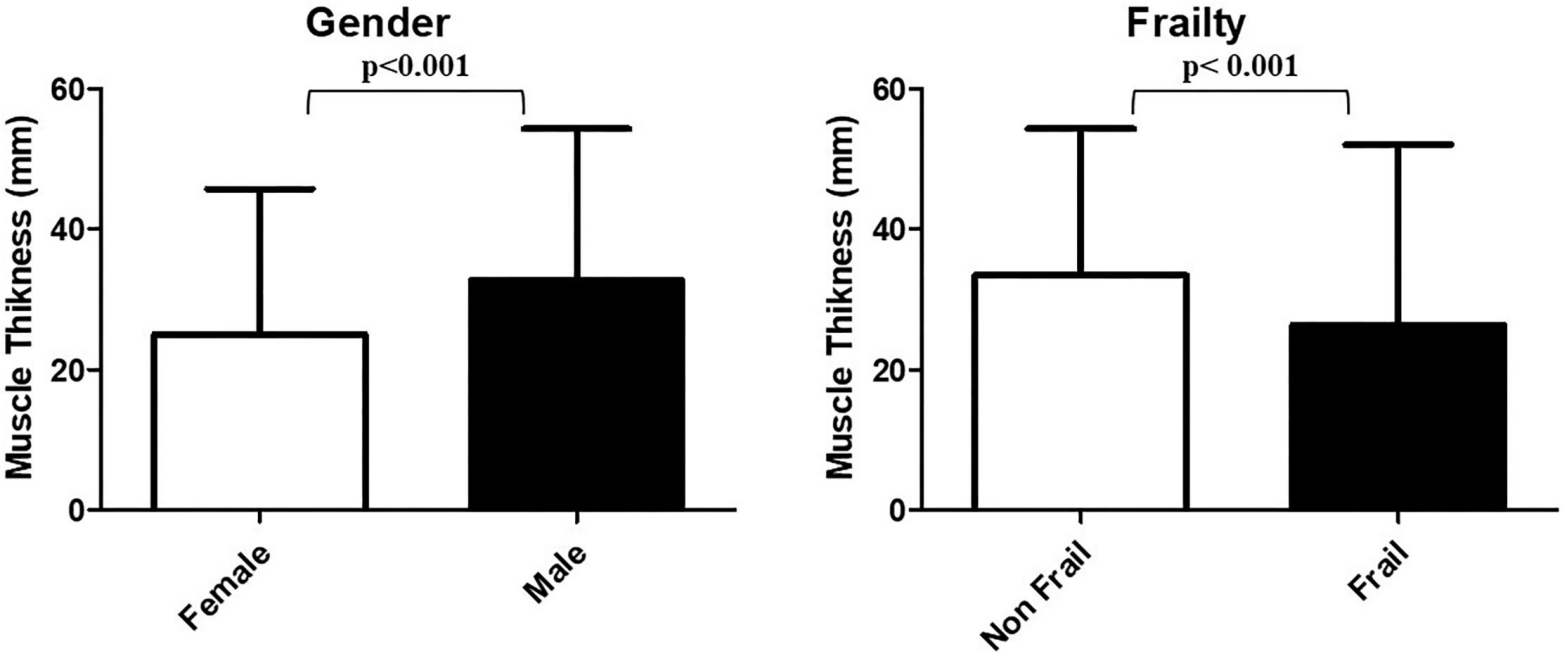
MT values stratified according to gender and frailty status. The *p*-values correspond to the Mann–Whitney *U*-test.

At univariate analysis, frail subjects resulted to be significantly older than non-frail ones [76 (71–82) vs. 73 (69–76) years, respectively, *p* = 0.007] and less predominantly male (58.2 vs. 75.6% *p* = 0.041). Of note, no other relevant differences emerged between the two groups in terms of BMI, kidney function, hemoglobin, and serum protein levels. As expected, frail patients presented worse scores in the great majority of tests and tools included in the CGA, compared to non-frail ones. While subcutaneous fat thickness did not statistically differ between the two groups, frail patients presented significantly lower thickness values of all examined muscles compared to non-frail ones, in particular MTs of rectus femoris plus vastus intermedius were 33.4 (26.8–38.5) and 26.4 (21.9–33) mm, respectively (*p* < 0.001). After stratification according to gender, the groups did not differ for age and BMI, but female participants showed higher subcutaneous fat thickness assessed through ultrasound, worse scores at physical performance tests (POMA, SPPB, and Grip Strength), and significantly higher FI [0.26 (0.2–0.42) vs. 0.38 (0.25–0.51), *p* = 0.012] (Supplementary Table S2). Consistently, all MUS thickness were significantly greater in male patients than counterparts [MT of rectus femoris plus vastus intermedius: 32.7 (24.6–37.9) mm and 25.0 (20.9–29.6) mm, respectively (*p* < 0.001)]. MT values stratified according to gender and frailty status are shown in Figure 2.

The multivariable regression analysis included as independent variables the binary predictor gender and the continuous predictors age, BMI, and MT of vastus intermedius plus rectus femoris (Table 4 and Supplementary Table S1). Importantly, the final model revealed that frailty was significantly and independently associated with age and MT (*p* < 0.01), while no relevant association emerged with BMI and gender. Notably, the contribution of MT to the overall R2 of the employed model was remarkable and superior to chronological age (55.07 vs. 44.93%, respectively). Similar results were obtained by replacing the independent variable BMI with subcutaneous fat (not shown).

**Table 4:** Regression analysis for frailty index.

**Variables**	**FI *R*^2^: 0.16**			
	**Coeff**.	**SE**	**Sig**.	**Partial contribution** **to *R*^2^ (%)**
Age	0.005	0.002	0.01	44.93
MT	−0.005	0.002	0.01	55.07
Gender	−0.031	0.032	0.333	–
BMI	0.001	0.003	0.753	–

*BMI, body mass index; FI, Frailty index; MT, muscle thickness (vastus intermedius plus rectus femoris)*.

## Discussion

The main result of the present study is represented by the identification of a significant association between frailty, assessed through accumulation of deficits model, and MT of vastus intermedius and rectus femoris, measured using MUS, in a population of older patients. To the best of our knowledge, this is the first study demonstrating a role of bedside MUS as marker of frailty evaluated through the CGA-based FI.

Along with the increasing interest of scientific community on sarcopenia, MUS has been proposed as valuable potential diagnostic tool to perform estimation of muscle mass. Compared to the current gold standard [Dual-energy X-ray absorptiometry (DXA), Computed Tomography (CT), Magnetic Resonance Imaging (MRI)], MUS represents a promising portable, accessible, cheap, cost-effective, and non-invasive imaging tool, particularly suitable for assessing older adults (12). Bioelectrical impedance analysis (BIA), which is also applicable bedside, is dependent on hydration status thus resulting less accuracy in specific clinical cases, as peripheral edema.

Several previous studies reported MT measurements of lower extremities, obtained through MUS, to be positively correlated with muscle strength and sarcopenia in older subjects (13). The reliability and validity of MUS in quantifying muscle size have been confirmed by the analysis of several studies of comparison with DXA (14), CT (15), MRI (5), especially in large muscle groups, as femoral quadriceps, whereas this ultrasound based technique may result challenging in the assessment of small muscles, probably caused by limited spatial resolution (5). The main problem with the use of MUS in the evaluation of muscle in the older patient is the little consistency, due to the lack of standardization in the adopted protocols, as emerged from literature search (4). Accordingly, the SARCUS working group has recently provided indications for an ultrasound protocol in the skeletal muscle assessment (10) from which the MUS method of the present study has been derived.

Frailty is a geriatric syndrome widely investigated in landmark studies through several valid models of assessment. Irrespective of the adopted tools, frailty has been associated with adverse health outcomes in different settings of care, thus increasing the scientific interest of geriatric research. Progressively more clinical decision processes are considering frailty status when selecting people to the most appropriate procedure (e.g., aortic valve replacement) or drug treatment (16). Among the multitude of instruments employed in geriatric medicine to measure frailty, FI seems to be the most suitable one to evaluate outcomes. Indeed, it is strongly associated with the risk of death and it may be considered an estimation of biological aging, which is more precisely correlated with morbidity and mortality than chronological age (17). Moreover, FI allows an accurate evaluation of physiologic reserve, that is known to exert an extremely important role in the response to stressors (18).

Following these premises, the aim of the present study was to evaluate whether the measurements of MT, obtained through an imaging technique increasingly employed in clinical research for sarcopenia assessment (which represents a physical substrate of frailty), were correlated with frailty, assessed through validated instruments of CGA, in a population of hospitalized older adults. The central hypothesis was that MUS may constitute an additional imaging tool of the geriatric multidimensional CGA-based approach.

The MUS values of MT of vastus intermedius plus rectus femoris came out to be significantly and independently correlated to FI in the study population, as emerged both at univariate and multivariable analyses. Importantly, the results of the latter analyses were corrected for potential confounding factors, such as BMI, gender, and age, aiming to avoid that such biological and anthropometric measures could influence the result and consequently condition MT impact on frailty. Furthermore, taking into account of the redistribution of body adiposity with age (19), we have also performed additional regression analyses introducing subcutaneous fat, instead of BMI, as independent variable, obtaining comparable results. Indeed, in both models, MT of vastus intermedius and rectus femoris remained independently correlated to the FI, as well as the chronological age, thus suggesting a potential role of MUS as instrumental domain of CGA. Of note, the final model has included MT and age, whose partial contribution to the global R2 was, respectively, 55.07 and 44.93%. This result is particularly interesting because it supports the robust contribution of MUS measures to explain the variability of the multifactorial CGA-based FI observed in the study population of older subjects, even when corrected by chronological age, which is an intrinsic characteristic of aging. Our results are in line with consolidated evidence indicating that female participants in clinical studies are frailer than male ones (20). We also confirmed the previous results showing that MT values of vastus intermedius plus rectus femoris obtained through MUS are significantly higher in male participants, while female individuals show the greater subcutaneous fat thickness (11).

Previous studies have focused on MUS as measure of frailty, with a specific interest on muscle strength and sarcopenia. The research group of Miron-Mombiela has demonstrated both MT and echo intensity of quadriceps to be correlated with grip strength in a subpopulation of adult outpatients aged 60 years and older. Moreover, the authors reported these measures to constitute imaging biomarkers of frailty, assessed according to Fried's criteria (21). A very elegant study by Narici and collaborators recently proposed the ultrasound sarcopenic index (USI) as novel imaging marker of reduced muscle mass associated with sarcopenia, independent of sex, body mass, and height that can impact on muscle sizes and architectural values. The authors calculated USI as the ratio between vastus lateralis muscle fascicle length and thickness, and reported that the greatest differences, compared to young controls, were found for the “mobility impaired elderly” and “sedentary elderly” groups (22). Another study, analyzing bedside MUS as a tool for sarcopenia assessment, has reported rectus femoris cross-sectional area to provide a prediction of adverse outcomes, as well as frailty diagnosed by FI, in the surgical intensive care unit (23).

Besides these pieces of literature that are consistent with the findings of the present study, our results are not in line with the previous evidence reported by Madden and collaborators, which performed point-of-care MUS of vastus medialis to test for association between MT and frailty in older adults. The authors detected only a weak correlation of MUS measurements with frailty, assessed through the Frailty Phenotype and the Clinical Frailty Scale (CFS), a 9-point judgment-based measure of frailty (24). Otherwise, it is important to mention that there are many and relevant differences in the applied protocols, including differences in the examined muscles. Although a gold standard methodology for MUS has not yet been established, also with regard to the anatomical muscles to be analyzed, we chose to measure the rectus femoris and the vastus intermedius MT based on the previous reports (11), because this approach offered the possibility of combining the measurements of two contiguous components of the same muscle group. Furthermore, from the pioneering studies on MUS by the research group of Abe, it has been developed the concept of “site-specific sarcopenia” to highlight that the age-related decline in muscle mass does not homogeneously proceed in all anatomic regions (25). Accordingly, it has been suggested that the muscle mass decline of rectus femoris seems to precede the one of other muscle groups (4, 26). Another main distinction between the two studies regards frailty assessment. Although the correlation between the two scales has been demonstrated to subsist, the CFS and the CGA-based FI present several relevant differences. As suggested by some authors, CFS is a valid instrument for initial frailty assessment, but it owns some limitations, in particular, in patients with dementia (27). Further, FI constitutes a more discriminative instrument compared to CFS, which is burdened by the rater subjectivity of clinical judgment (28).

Thus, taking into account the multifactorial nature of FI and considering the great heterogeneity which characterizes older subjects, the promising results of the present study allow to speculate on the potential role of MUS in detecting phenotypic characteristics of aging other than those canonically captured by the consolidated CGA tools.

## Limitations

The study participants were recruited from a single geriatric medicine clinic, a population that tends to be frailer than the general population, due to multiple chronic illnesses. No control group was included in the protocol. The sample size calculation is burdened by the lack of evidence and reference values in the method and by the specificity of the population in question. Larger studies are needed to confirm our findings, even considering MT controlled by definite physical indicators which may affect its measures, not yet established by the scientific community. Even though the comparison with sarcopenia was not an aim of the present research, the lack of ascertained diagnosis of muscle mass decline does not allow a comparison between the MT measurements and the result of other reference methods.

## Conclusion

Frailty is a multifactorial geriatric syndrome; CGA-based tools are valid instruments for its diagnosis and management. MUS measures of MT of vastus intermedius plus rectus femoris resulted to be significantly correlated to FI in a population of hospitalized older patients, independently from other considered covariates. Further studies are needed to confirm this association and determine the clinical impact of these findings, aiming at defining MUS as an additional imaging domain of frailty.

## Data Availability Statement

The original contributions presented in the study are included in the article/Supplementary Material, further inquiries can be directed to the corresponding authors.

## Ethics Statement

The studies involving human participants were reviewed and approved by Comitato Etico per le attività biomediche - Università degli Studi di Napoli Federico II. The patients/participants provided their written informed consent to participate in this study.

## Author Contributions

LB and GR conceived this study. LB, FP, and CR extracted the data. LB, DV, and KK designed and performed the statistical analyses. LB, LF, FR, IS, GG, and GF wrote the first draft of the manuscript. NF and GR reviewed and modified the final manuscript. All authors read, critically reviewed, and approved the final manuscript.

## Conflict of Interest

The authors declare that the research was conducted in the absence of any commercial or financial relationships that could be construed as a potential conflict of interest.

## Publisher's Note

All claims expressed in this article are solely those of the authors and do not necessarily represent those of their affiliated organizations, or those of the publisher, the editors and the reviewers. Any product that may be evaluated in this article, or claim that may be made by its manufacturer, is not guaranteed or endorsed by the publisher.
