# Supplementary material for: Muscle Ultrasound as Imaging Domain of Frailty
Source: Front Med (Lausanne). 2022 Jul 11;9:922345. doi: 10.3389/fmed.2022.922345 (PMC9309884; doi:10.3389/fmed.2022.922345)
Supplement: Supplementary file 1 [file Data_Sheet_1.docx]

**Table S1.** Characteristics of the population stratified according to gender.

| **Characteristics** | **Male**  **(n=87)** | **Female**  **(n= 49)** | **Sig.** |
| --- | --- | --- | --- |
| Age *(years)* | 73 (69-80) | 76 (71-82) | 0.063 |
| BMI *(Kg/m^2^)* | 26.23±4.00 | 25.61±5.31 | 0.440 |
| Haemoglobin *(g/dL)* | 12.14±2.46 | 11.69±2.37 | 0.313 |
| eGFR *(mL/min/1.73m^2^)* | 66.5 (47-82) | 62 (45-81) | 0.4714 |
| Serum protein *(g/dL)* | 6.47±0.75 | 6.45±0.74 | 0.863 |
| MMSE *(/30)* | 25.5 (22.5-27) | 24.5 (20.5-26.5) | 0.184 |
| BADL *(/6)* | 6 (5-6) | 5 (4-6) | 0.003 |
| IADL *(/8)* | 7 (4-8) | 6 (3-8) | 0.096 |
| POMA *(/28)* | 26 (20-28) | 21 (13-25) | <0.001 |
| SPPB *(/12)* | 6.66±3.57 | 4.43±2.89 | <0.001 |
| MNA *(/30)* | 22.5 (19.5-25) | 21 (18-24) | 0.104 |
| CIRS *(n)* | 3.85±2.11 | 3.59±1.68 | 0.463 |
| Chronic drugs *(n)* | 6.90±2.84 | 6.39±2.84 | 0.319 |
| PASE *(n)* | 86 (40-130) | 59.3 (17.5-123) | 0.179 |
| Social Support Score *(/17)* | 6.15±2.73 | 7.04±2.62 | 0.066 |
| Grip Strength *(kg)* | 26.89±9.40 | 16.71±7.54 | 0.017 |
| FI *(/1)* | 0.26 (0.2-0.42) | 0.38 (0.25-0.51) | 0.012 |
| Rectus femoris *(mm)* | 18.24±4.79 | 14.82±3.47 | <0.001 |
| Vastus intermedius *(mm)* | 13.2 (9.8-16.9) | 10.6 (8.1-14.2) | 0.024 |
| MT *(mm)* | 32.7 (24.6-37.9) | 25 (20.9-29.6) | <0.001 |
| Subcutaneous fat *(mm)* | 9.4 (6.6-13) | 16 (12.5-23.5) | <0.001 |

BADL, Basic Activity of Daily Living; BMI: Body Mass Index; CIRS, Cumulative Illness Rating Scale; eGFR, estimated Glomerular Filtration Rate (according to CKD-EPI formula); FI, Frailty Index; IADL, Instrumental Activity of Daily Living; MMSE, Mini Mental State Examination; MNA, Mini Nutritional Assessment; MT, Muscle Thickness (vastus intermedius plus rectus femoris); PASE, Physical Activity Scale for the Elderly; POMA, Tinetti’s Performance Oriented Mobility Assessment; SD, Standard Deviation; SPPB, Short Performance Physical Battery.

**Table S2.** Association of MT and age with FI (Linear regression analysis)

|  | **Frailty Index** | | | |
| --- | --- | --- | --- | --- |
|  | ***Unadjusted*** | | ***Adjusted**** | |
|  | ***Coeff. (SE)*** | ***p-value*** | ***Coeff. (SE)*** | ***p-value*** |
| **Muscle Thickness** | -0.07 (0.01) | <0.001 | -0.06 (0.01) | 0.001 |
| **Age** | 0.07 (0.02) | <0.001 | 0.07 (0.02) | 0.001 |

*Multivariable-adjusted model on Body Mass Index and gender.

**Simple size calculation.** The sample size was estimated on the basis of previous evidence on the method employed (muscle site and ultrasound assessment) (11), considering a prevalence of frail hospitalized patients double that of non-frail ones, with an expected difference in muscle thickness of about 12% between the two groups. Establishing a type I error of 5% and a statistical power of 80%, at least 108 patients were needed (no less than 72 frail).
